# The impact of an intervention program on students’ spatial reasoning: student engagement through mathematics-enhanced learning activities

**DOI:** 10.1186/s41235-018-0147-y

**Published:** 2018-12-26

**Authors:** Tom Lowrie, Tracy Logan, Danielle Harris, Mary Hegarty

**Affiliations:** 1 University of Canberra, Canberra, Australia; 2 University of California Santa Barbara, Santa Barbara, CA USA

**Keywords:** Spatial reasoning, Spatial skills, Intervention, Middle school, Classroom research

## Abstract

**Background:**

Spatial reasoning skill has consistently been found to be malleable. However, there is little research to date on embedding spatial training within learning frameworks. This study evaluated the effects of a classroom-based spatial reasoning intervention on middle school children’s spatial reasoning. Participants included 337 students from 15 classrooms across 6 schools with 8 experimental and 7 control classes. The program was designed for grades 3, 4, 5, and 6. The intervention program was delivered within the Experience-Language-Pictorial-Symbolic-Application (ELPSA) framework and was delivered across 10 weeks by classroom teachers, while the control group received standard mathematics instruction.

**Results:**

Children in the experimental classes outperformed the control classes on spatial reasoning at the conclusion of the program. The intervention program received high levels of engagement and evidence for development through the stages of the ELPSA framework.

**Conclusions:**

This paper provides evidence for the effectiveness of a rich spatial training program delivered within a learning framework. This program has applications for spatial thinking in Science, Technology, Engineering and Mathematics.

**Electronic supplementary material:**

The online version of this article (10.1186/s41235-018-0147-y) contains supplementary material, which is available to authorized users.

## Significance statement

The field of spatial reasoning research is receiving a great deal of attention due its strong links to Science, Technology, Engineering and Mathematics (STEM) and the inherently spatial nature of our world. Previous work in spatial training has been limited to researcher-driven studies. The significance of the present study is found in the application of spatial training by classroom teachers with a great deal of success across four middle school grades with children of all ranges of pre-training spatial skill. The participants in the study reported high levels of engagement in the program and enthusiasm for the applications of the work.

## Introduction

Spatial reasoning has consistently been linked to success in STEM outcomes and lifelong STEM career achievement (Kell, Lubinski, Benbow, & Steiger, 2013; Lean & Clements, 1981; Nath & Szücs, 2014; Wai, Lubinski, & Benbow, 2009). Historically, educational focus is often directly on pedagogical content related to reading, mathematics, and science. Spatial thinking has received much less attention (National Research Council, 2006). Given the established importance of spatial thinking, Uttal et al. (2013) suggest that early spatial skill intervention may increase students’ spatial competencies so they are not overwhelmed by STEM content knowledge in the later years of education (see also Newcombe, 2016).

Spatial thinking involves the understanding of three related properties: (1) an awareness of *space* itself, such as distance and dimensions; (2) the *representation* of spatial information (both in the mind and externally in graphics such as diagrams and maps); and (3) the *reasoning* involved in interpreting this spatial information and making decisions (National Research Council, 2006). In an instructional environment, effective teaching of spatial thinking must address and foster all three elements, with an emphasis on the cognitive processes themselves (DeSutter & Stieff, 2017), separate to pedagogical knowledge.

The current investigation describes an empirical study that was conducted to determine the effectiveness of an intervention program on developing primary-aged students’ spatial reasoning. Specifically, this paper considers the extent to which the intervention program is effective (1) across different age groups and (2) for students of different initial spatial skill levels. More holistically and qualitatively, we determine the extent to which students engaged with learning activities within and across the respective components of the learning design.

### Malleability of spatial reasoning

As an ability to represent and manipulate symbolic or nonlinguistic information, spatial reasoning is believed to be malleable and transferable across the lifespan (Linn & Petersen, 1985; Terlecki & Newcombe, 2005) and enhanced through training (see Uttal et al., 2013 for a review). Moreover, improvement on spatial tasks is found to be transferable to novel stimuli within the same task or to other spatial tasks (e.g., Wright, Thompson, Ganis, Newcombe, & Kosslyn, 2008). In their meta-analysis Uttal et al. (2013) concluded that there is solid evidence that spatial reasoning skills can be trained, with a mean effect size of .47 for improvement in spatial training across studies.

There is evidence for the development of spatial reasoning skills with age (Newcombe & Huttenlocher, 2003; Piaget & Inhelder, 1956). Previous work by Lowrie, Logan, and Ramful (2017) reported on the effectiveness of a spatial training program for children in grades 5–6 in both their spatial reasoning and mathematics performance. In the present paper we include new data on the implementation of the spatial training with grades 3 and 4 students, in addition to the previously published data for grades 5 and 6, to examine the effectiveness of training across a wider age range. In their meta-analysis, Uttal et al. (2013) found no significant differences in the effectiveness of spatial training across age groups, but they noted that there were very few studies of spatial training in children younger than 13 years. The first aim of this study was to determine whether the intervention program was effective with students of different ages.

### The effectiveness of an intervention program for participants of varying spatial skill

Uttal et al. (2013) cite the initial level of spatial performance as a contributing factor in an intervention program’s success. Although only 19 of 206 studies used a screening agent to specifically target low spatial scorers, these studies reported significantly larger effect sizes than the remaining studies. There is evidence of an initial lag in the learning of low spatial participants, who show different improvement trajectories compared to higher performing students (Terlecki, Newcombe, & Little, 2008). After week 6 of Terlecki and colleagues’ mental rotation training program, the low spatial learners got over their initial “hump” and progressed at a comparable rate to that of the high spatial learners. The present intervention extended beyond 6 weeks and did not identify or initially target low performing students since the research design required implementation in whole-class contexts. Nevertheless, the second objective of this study, a novel contribution of the present paper (including new analyses of the grades 5–6 data), was to determine whether the intervention was more effective for students of varying spatial skill.

### Components of spatial reasoning skill

Researchers have identified a number of factors constituting spatial reasoning skill; however, to date there is no consensus in its exact structure or consistency in measurement within the literature (Hegarty & Waller, 2004). One of the challenges in assessing the multidimensionality of spatial reasoning is the nature of the assessments themselves. In general, the psychometric measures that are used to define the subconstructs contain markers that explicitly measure the related factor (Kozhevnikov & Hegarty, 2001; Linn & Petersen, 1985). This circular research design does not lend well to robust measures and theoretical development. In addition, many studies employ adult tests of spatial reasoning on child populations without validating their use (Newcombe, 2016).

The spatial training program reported in Lowrie et al. (2017) and subsequently assessed in the present study used a measure of spatial reasoning, the Spatial Reasoning Instrument (SRI), based on the national school curriculum standards in the corresponding population (Ramful, Lowrie, & Logan, 2017). Importantly, the SRI provides broad coverage of three subconstructs of spatial reasoning that have been well established in the research literature (Linn & Petersen, 1985; Lohman, 1979; McGee, 1979), namely mental rotation, spatial orientation, and spatial visualization. This afforded the opportunity to conduct analyses on the effectiveness of the intervention program on students with different levels of spatial reasoning skill with rigor and depth.

#### Mental rotation

Mental rotation is the ability to accurately rotate a two-dimensional (2D) shape or a three-dimensional (3D) object in the “mind’s eye” in order to perform a subsequent task, such as the comparison tasks devised by Shepard and Metzler (1971). It is an object-centered transformation that is detached from the viewer and requires no change in perspective (Carlson-Radvansky & Radvansky, 1996). Mental rotation has been extensively researched due to its strong association with success in STEM (e.g., Cheng & Mix, 2014; Von Károlyi, 2013). Mental rotation is believed to be malleable as a result of experience and learning (Peters et al., 1995; Stransky, Wilcox, & Dubrowski, 2010).

#### Spatial orientation

Spatial orientation requires an egocentric transformation of imagining a change in one’s own perspective. This ability can be dissociated from mental rotation (Hegarty & Waller, 2004; Kozhevnikov & Hegarty, 2001); however, the use of either a rotation or re-orienting strategy can stem from the content of the task. The dominant strategy is view re-orientation when a perspective change of more than 90 degrees is required (Kozhevnikov & Hegarty, 2001); in the SRI all spatial orientation tasks demanded a perspective change of greater than 90 degrees.

#### Spatial visualization

Of the three constructs, spatial visualization is the least well defined by theoretical frameworks; rather it is often defined by the tests used to measure it (Hegarty & Waller, 2004; Kozhevnikov & Hegarty, 2001). Furthermore, much of the distinction between spatial visualization and mental rotation is in the complexity of the spatial transformations to be imagined. In mental rotation the object remains intact as it moves through space, while spatial visualization addresses the transformations *within* the object (Sorby, 1999). In the present study, spatial visualization is defined as the ability to mentally transform or manipulate the visuospatial properties of an object, distinct from rotation of the object (i.e., mental rotation) or varying one’s perspective (i.e., spatial orientation), for example, visualizing a cube from its net or predicting a pattern on a piece of paper that has been unfolded. In a study across three grade levels, Mix et al. (2016) and colleagues found traditional tests of spatial visualization to be strong predictors of mathematics performance.

## Conceptual underpinnings of the study

In the research to date, most spatial reasoning intervention programs have been administered by a member of the research team with the training neither presented within whole-class contexts nor situated within the participants’ standard classroom practices. The present intervention program (introduced by Lowrie et al., 2017) was designed within a pedagogical framework that ensured participants’ classroom teachers could administer the program. As part of our new analysis of data from this study and our extension of this study to younger children (grades 3 and 4), we aimed to determine whether student engagement was evident across all components of the pedagogical design (our third objective).

The intervention program was designed within a framework that drew on well-regarded sociological and psychological understandings of learning (Adler, 1998; Cobb, 1988; Lerman, 2003). We utilized the Experience-Language-Pictorial-Symbolic-Application (ELPSA) learning framework (Lowrie & Patahuddin, 2015) in order to design the lessons for the spatial reasoning intervention program and explain how students developmentally understand concepts within the respective spatial reasoning constructs. The framework promotes learning as an active process in which individuals construct their own ways of knowing (developing understanding) through discrete, scaffolded activities and social interactions. Lowrie and Patahuddin maintained that each step of the framework was critical for establishing sense making, and that the sequence provides a logical structure to scaffold, reinforce, and apply knowledge and concepts.

The first component of the learning framework (Experience) draws on the knowledge that students possess. In this stage the teacher should determine what the students know and what new information needs to be introduced to scaffold their understanding. The second component of the framework (Language) outlines how terminology is used to promote understanding. This stage of the process is also associated with particular pedagogical practices, since it is important for teachers to model appropriate terminology and encourage students to use this language to describe their understandings in ways that reinforce their knowledge and promote discourse with others. The third component of the learning framework (Pictorial) is characterized by the use of spatial and concrete representations to exemplify ideas and concepts (Burte, Gardony, Hutton, & Taylor, 2017; Pillay, 1998). Such representations could be constructed by the teacher (including shared resources and artifacts) or students (including drawing diagrams or visualizing). The fourth component (Symbolic) is aligned to the formalization of ideas or concepts. This stage draws on students’ capacity to represent, construct, and manipulate analytic information with flexibility and a degree of fluency (Stieff, 2007). The final component of the learning framework (Application) highlights how symbolic understanding can be applied to new situations. This is evident in students’ ability to transfer their knowledge to novel situations.

## Present study

The present paper examines the detail and scope of the intervention program (aim 3) in the 9–13 age range (grades 3 to 6). Specifically, the study focused on the effectiveness of program instruction in relation to the impact of the intervention (1) on students’ spatial reasoning performance and (2) on students of different initial skill level. The study also examined student engagement in relation to (3) the extent to which the respective elements of the program’s learning cycle (ELPSA) evoked spatial reasoning.

## Method

### Participants

A total of 337 students from six primary schools in Canberra, Australia participated in the study. There were 193 students (91 female) from eight classes who participated in the experimental condition (intervention) and 144 students (83 female) from seven classes who participated in the control condition. The schools were distributed throughout the Canberra region and covered a broad socio-economic demographic, with ICSEA^1^ scores ranging from 996 to 1194. Table 1 shows descriptive statistics for the most relevant teacher and classroom characteristics separated by condition.

**Table 1:** Descriptive statistics for teacher, classroom, and student characteristics

	Total sample	Intervention	Control
*N* (students)	337	193	144
Years of teaching experience: range	1–23	1–19	1–23
Years of teaching experience: *M (SD)*	9.33 (*7.06*)	8.90 (*6.45*)	9.88 (*8.18*)
Class size: *M*	24.07	24.13	24
Student age: *M (SD)*	11.61 (*.59*)	10.96 (*1.07*)	10.39 (*0.83*)
Student gender (% female)	51.6	47.2	59.3

There were no significant differences in teaching experience between the two conditions; *t*(16) = .28, *p* = .78

*M* mean, *SD* standard deviation

### Materials

#### Spatial Reasoning Instrument

**Fig. 1:**
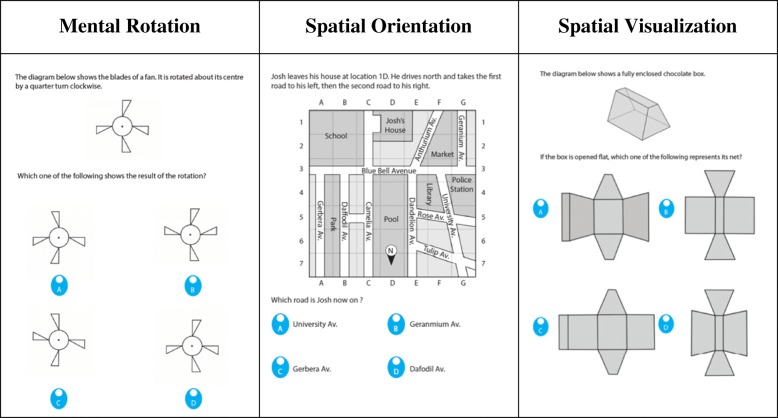
Sample items from the SRI (Ramful et al., 2017)

The paper-and-pencil Spatial Reasoning Instrument (SRI; Ramful et al., 2017) was developed specifically for primary school children, based on three constructs (with an equal number of items per construct): mental rotation, spatial orientation, and spatial visualization. The three subscales have strong correlations with measures of these constructs in the cognitive psychology literature (Ramful et al., 2017). Scores on the SRI were the total number of correct items for each participant; unanswered items were assigned a score of 0. Examples of the items are presented in Fig. 1. The 30 items used in the present study were drawn from the pool of items used to construct the SRI. The test items were common across all year levels. Cronbach’s alpha for the 30-item test was .81.

### Procedure

In order to recruit teachers, school principals in the authors’ network across the state were contacted. The study ran in the second half of the 2015 school year.^2^

#### Professional development workshop

Teachers in the intervention condition participated in a 5-day professional development workshop (as described by Lowrie et al., 2017) to develop the spatial reasoning intervention during the Australian winter term at the authors’ university. During this workshop, intervention teachers became familiar with the spatial reasoning constructs and pedagogical framework of the lessons. The teachers were encouraged to adapt the lesson plans to accommodate their personal pedagogical strengths, classroom culture, and student needs. Nevertheless, they were required to ensure that they delivered all content and learning activities described in the 20 lessons.

At the completion of the workshop, the intervention program teachers were equipped with detailed lesson plans, teaching materials (including concrete manipulatives), digital resources accessed through tablet apps, and iPads if they were not readily accessible in their schools. The teachers also received electronic copies of the development sessions to help them present the lessons based on the theoretical underpinnings prescribed in the program.

#### Content of the spatial reasoning program

The intervention was implemented over 10 weeks during twice-weekly 60-min class periods. Table 2 provides a summary of the learning activities presented in the intervention program. The intervention replaced the measurement and geometry units that would usually be taught from the Australian Curriculum—the units most likely to engage students in spatial thinking. Each spatial construct had a dedicated 6 h of teaching over 3 weeks. The final 2 h in the last week of the program combined the topics from the previous 9 weeks.

**Table 2:** Structure of the intervention lessons

Week	Construct	Specific topics
1	Mental rotation	2D rotation
2	Mental rotation	3D rotation
3	Mental rotation	Rotational symmetry (symmetrical patterns)
4	Spatial orientation	Drawing and navigating maps Interpreting map conventions
5	Spatial orientation	Orientation around a cardinal point Reading inverted maps
6	Spatial orientation	Perspective taking Alternate views
7	Spatial visualization	Paper folding and cutting
8	Spatial visualization	Nets of solids
9	Spatial visualization	Reflection and symmetry Hidden blocks and cross sections of 3D objects
10	Integration	Teacher’s choice

If only one topic is listed for a week, then both lessons were on that topic for the week

During the intervention program, the students were exposed to learning activities that encouraged spatial thinking. The tasks were not “stand alone” spatial training tasks (such as those used by Cheng & Mix, 2014). Rather, the tasks were embedded into activities that fostered spatial thinking (Newcombe, 2017), including open-ended tasks that could be solved with multiple solutions. Intervention students were introduced to learning experiences that evoked spatial reasoning through inquiry-based engagement—through both individual and cooperative-based experiences situated within the ELPSA pedagogical framework, as detailed in the following paragraph. By way of example, in a mental rotation lesson, students drew objects to represent 2D shape and 3D object rotations. As their expertise developed, they were encouraged to visualize these transformations and not rely on concrete support. The spatial orientation tasks required the students to draw maps that would allow others to navigate from school to their home, with maps including directions that utilized compass points and rotational terminology. The tasks required students to decode maps of different orientations, including maps where the “North” compass direction was atypical (i.e., not at the top of the page). Spatial visualization activities encouraged students to use isometric and grid paper to represent 3D objects and determine the number of blocks within 3D objects. In addition, students used imagery to imagine how nets could be folded and unfolded from 3D objects. Across all lessons, students were encouraged to use visualization strategies to make predictions as part of their learning process rather than relying solely on concrete materials.

The six lessons within each construct were framed around the ELPSA learning framework (Lowrie & Patahuddin, 2015). An example of how the ELPSA framework was embedded into the conceptual development of one lesson of the spatial visualization construct (Reflection and Symmetry, introduced in Week 9) is presented in Additional file 1. The Experience component of the lesson encouraged students to consider familiar reflections and describe the contexts in which these reflections are found (e.g., well-known branding symbols like the golden arches or designs in architecture). Students drew these reflections along both *y* and *x* axes. The Language component encouraged the explicit use of reflection terminology (e.g., line of symmetry, reflection). The Pictorial phase encouraged students to engage with more difficult concepts with the support of concrete manipulatives or the encoding of information—in the Reflection and Symmetry lesson this involved ideas associated with reflection along diagonals. For example, the students were encouraged to visualize what the reflection would look like and then use materials (e.g., a mirror) to check their hypothesis. As they became more proficient, they were encouraged to rely less on the materials and utilize internal visualization processes only. In the Symbolic phase, students were encouraged to establish patterns and relationships that drew on analytic reasoning, such as an understanding of perpendicularity. The final component was the Application of knowledge, where analytic thinking was applied to new situations. In the example presented in Additional file 1, the student showed the reflection of a bus projected from water on a road.

The classroom teachers were encouraged to verbalize their thinking, and that of their students, through modeling and scaffolding. They were also encouraged to “overemphasize” spatial arrangements in the classroom and the school environment. The lessons within the program encouraged participants to verbalize their thinking and justify understanding in terms of real-world contexts and applied examples. A diverse range of activities were chosen so that children could begin to develop a more flexible, spatial thinking approach when encountering novel situations (Reys, Lindquist, Lambdin, & Smith, 2009).

#### Control group

The control group’s learning activities were drawn from the Australian Curriculum guidelines (ACARA, 2015). In Australia, the school curriculum outlines the necessary content to be taught for each age group, but the school and classroom teacher determine the structure of the lessons. The content covered by the control group teachers included concepts associated with geometry and measurement, numbers and algebra, and statistics and probability. Any opportunity for development of students’ spatial reasoning skills would be covered in the geometry strand of the mathematics curriculum, in particular, content associated with “shape” and “location and transformation”. In grades 3 and 4, students make models of 3D shapes, create and interpret maps, use direction to interpret maps, and create symmetrical patterns. In grades 5 and 6, new content includes the use of grid references on maps and the introduction of the Cartesian coordinate system. To this point, the foundations for understanding shape, location, and transformation are established in grades 3 and 4.

#### Test administration

In each class, testing took place in the classroom during regularly scheduled school hours. The SRI was administered in a group setting by a member of the research team. The test was untimed but was completed by all students within 50 min. After a brief introduction, each child worked on an individual test booklet. Testing was completed within the 2 weeks prior to the commencement of the 10-week intervention (pre-test) and within 2 weeks of its completion (post-test).

## Results

The results of the study are presented in three sections. The first section presents the effect of the spatial reasoning program. Since the research design contained nesting structures of students within classrooms, a multilevel (hierarchical) modeling approach was adopted to analyze group differences on pre-tests and treatment gains. The second section analyzes the effect of the program in relation to student’s initial spatial rank. Hierarchical linear modeling (HLM) was again conducted, with a design that had students nested within classrooms at Level 1, with students’ pre-test scores used to determine initial spatial reasoning level. Moreover, using the pre-test as a covariate is highly recommended in such situations (Rausch, Maxwell, & Kelley, 2003). The third section provides a qualitative description of the program’s fidelity, specifically documenting the extent to which classroom teachers and their students represented spatial information within the ELPSA learning framework.

A preliminary analysis was undertaken to establish whether the cohorts (intervention vs. control) had similar initial spatial reasoning scores for both grades. There was no difference between the mean scores of the intervention and control groups on the SRI pre-test *t*(335) = .27, *p* = .79. Given the literature on gender differences in spatial reasoning (Linn & Petersen, 1985; Voyer, Voyer, & Bryden, 1995), analyses were conducted to determine whether gender differences were evident in the present sample. There were no significant gender differences in spatial skill found at pre-test, *t*(331) = 1.23, *p* = .22. Therefore, the analysis in the present study will focus on the effectiveness of the intervention program, independent of gender.

### Effectiveness of the spatial reasoning program

We analyzed group differences on pre-tests using a two-level HLM model (students within classrooms) with conditions dummy coded (1 = intervention and 0 = control). A two-level model was also used to analyze pre-test–post-test gains, with condition groups similarly dummy coded to determine the direct effects of the interaction. An intra-class correlation (ICC) was conducted on these data to determine the variability between and within the group clusters. The ICC measure was .102 with a 95% confidence interval from .031 to .256 [*F*(17,252) = 2.17, *p* < .001]. Although the design had a small number of groups for an HLM model, the ICC measure indicated sufficient power and a low degree of dependency on type I error (see Aarts, Verhage, Veenvliet, Dolan, & Van Der Sluis, 2014, for a description of the role of ICC in nesting designs). Additional file 2 provides an example of data analysis using HLM.

Results from the hierarchical linear models for pre-test–post-test gains revealed gain scores greater than 0 for each group across the two measures (see Table 3 for observed mean gains). The intercept slope was statistically significant, *F*(1, 11) = 54.16, *p* < .001. On average, students in the intervention group gained 1.35 score points more than the control group on the SRI *t*(12) = 11.25, *p* < .001.

**Table 3:** Pre-tests, post-tests, and pre-test–post-test gains for SRI by condition

Measure	Intervention						Control					
	Pre-test		Post-test		Gain		Pre-test		Post-test		Gain	
	*M*	*SD*	*M*	*SD*	*M*	*SD*	*M*	*SD*	*M*	*SD*	*M*	*SD*
SRI	15.24	4.75	19.48	5.51	4.42	4.95	15.09	4.77	18.76	5.41	3.67	4.95

### Effectiveness of the program in terms of individual differences

To determine the extent to which the intervention program advantaged students of varying levels of initial spatial reasoning performance, a subsequent analysis was undertaken to determine whether there was an interaction effect between students’ initial spatial reasoning skill and their post SRI scores. Students’ initial SRI scores were coded as low, medium, or high, where low equaled the lowest quartile, high equaled the highest quartile, and medium the remaining 50%. Gain scores were generated for the respective spatial ranks. Results from the hierarchical linear model showed gain scores greater than 0 for each group across the two measures (see Table 4 for observed mean gains). The 2 (control vs. intervention) × 3 (low, medium, high) analysis of variance (ANOVA) revealed no statistically significant interaction between cohort and initial rank on the SRI, *F*(2, 14) = 1.38, *p* > .05.

**Table 4:** SRI gain scores (mean (standard deviation)) by spatial rank for each cohort

Spatial rank	Intervention	Control	Effect size (*d*)
Low	4.52 (3.16)	3.13 (3.22)	.44
Medium	4.27 (3.47)	2.42 (3.11)	.56
High	4.23 (3.31)	2.90 (3.41)	.43

Intervention: Low *N* = 60, Medium *N* = 80, High *N* = 53. Control: Low *N* = 39, Medium *N* = 62, High *N* = 43

Effect sizes (Cohen’s *d*) between intervention and control group SRI gain scores by spatial rank are presented in Table 4. We can conclude that the spatial reasoning program was beneficial for the intervention group irrespective of their initial spatial reasoning skill.

### Student engagement within the pedagogical framework

During the 10-week intervention, student work samples were collected and analyzed to determine levels of fidelity and student engagement within the ELPSA learning cycle. Additional file 1 describes learning activities that were aligned to the five components of the ELPSA model within spatial visualization, contextualized within the Week 9 Reflection and Symmetry activities. The first column represents ideas and activities presented by classroom teachers, including artifacts used to introduce activities within the ELPSA framework, providing evidence that the classroom teachers presented activities and learning ideas within the respective components of the ELPSA framework.

The teachers began the topic from the viewpoint of what students knew about the topic and encouraged active engagement through contextualized whole-class discussions (Experience). They were explicit about the terminology used—increasing the complexity of the language conventions throughout the topic—and encouraged students to reflect upon the relevance of this language at the completion of the lessons (Language). In the Pictorial phase, the teachers modeled symmetry concepts through diagrams and encouraged students to do the same, aiding the transition from concrete and diagrammatical representations to more sophisticated visualization strategies. The teachers then encouraged students to reason analytically, as a transition beyond representing information “in the mind’s eye.” This symbolic reasoning was evident in the development of rules such as the orientation of objects after a diagonal reflection. Finally, the teachers presented open-ended activities that required students to apply concepts to other situations (Application).

The second column of Additional file 1 highlights student work samples within each of the five ELPSA components. The work samples align well with how the teachers modeled conceptual development throughout the unit—highlighting the fidelity of the program. These lessons required the students to encode during each component of the framework as they moved from concrete, to visual, to analytic reasoning. The encoding techniques supported students’ learning as they reflected upon and evaluated their reasoning.

The third column provides examples of student reflections in their own voice as they progressed through the spatial visualization activities of the program. These student reflections highlight the movement toward analytic thinking. The notion that “symmetry is something like butterfly wings” indicates the establishment of context. The language conventions of symmetry are made explicit in comments like “how to flip it on the *y* or *x* axis so I was trying to visualize the mirror,” providing evidence for the alignment of everyday ideas to mathematics terminology. At the Pictorial phase, students displayed a decreasing need for concrete manipulatives in solving tasks, for example “picturing it in my mind and trying to think of how the page was folded diagonally.” At the analytic stage the students used gesture to support their problem solving before progressing to more complete understandings of perpendicularity “I was imagining a mirror on the fold of the page, using visual measurements to make it as accurate as possible.” Instances of application were less common; however, detail and accuracy become more commonplace.

The process of concept development was established initially from a shared understanding of contextual knowledge (Experience and Language) and supported through concrete materials and gesture, the encoding of information, and the opportunity to internally visualize (Pictorial). From this point, competent students progressed toward analytic thinking (Symbolic and Application). That is, all students participated (engaged) in spatial visualization learning activities within the first three components of the learning framework. It is not surprising that we found less evidence of engagement in the more analytic components (especially at the Application level).

## Discussion

We examined the impact of a spatial training intervention program based on the ELPSA theoretical framework and implemented by teachers in their own classrooms. There were large gains in spatial reasoning from pre-test to post-test, with statistically significant differences between intervention and control groups. Moreover, there was strong fidelity evidence to suggest that both the teaching activities and student engagement progressed through the respective stages of the ELPSA framework. Interestingly, the control groups also improved, potentially due to the practice effect in retaking the test (Uttal et al., 2013) and the nature of their standard geometry curriculum, but critically, the intervention group improved more than the control groups, and this effect was significant. Uttal et al. (2013) highlighted the need for more research regarding how spatial understanding progresses in the middle years of school—this study demonstrated that the intervention program was effective for classrooms that spanned four grade levels.

### Growth based on initial spatial reasoning

Spatial reasoning scores for students in the intervention group improved at consistent rates irrespective of initial spatial reasoning score, compared to the control group. Students initially classified with either low, moderate, or high spatial reasoning had moderately high effect size gains—with each cohort achieving greater than *d* = .4 higher than the comparable control group. The personalized nature of the ELPSA framework, where all students are encouraged to move through the learning cycle in a learner-focused and constructivist manner, might have contributed to these outcomes. In order for students with higher levels of spatial proficiency to also benefit from the training program, it is necessary for these students to engage in symbolic reasoning and more sophisticated levels of pattern abstraction (Jurdak & El Mouhayar, 2014; Landy, Allen, & Zednik, 2014). Within the ELPSA framework, such engagement is promoted in the Symbolic and Application components in particular (Lowrie & Patahuddin, 2015). The Application of spatial thinking developed through the program has the potential reach into STEM practices (DeSutter & Stieff, 2017). Modifications to the program might need to ensure that teachers expose higher performing students to learning activities within the Symbolic and Application components sooner.

### Implementation of the pedagogical framework (ELPSA)

The ELPSA framework became a point of reference for how the classroom teachers introduced learning activities and how the students acquired conceptual understanding. Thus, the teaching artifacts provided insights into the fidelity of the instruction, while the student work samples informed our understanding of how students made sense of the activities. In addition, the framework became a point of reference for our capacity to reflect upon the strength of the intervention design. Consequently, we maintain that the classroom-based intervention program should be developed within a pedagogical framework.

### Future directions, limitations

Although this investigation has established the success of the intervention program, future work should investigate the extent to which this success is derived from students’ exposure to the learning activities and/or the embedding of the activities into the ELPSA framework. We have evidence that the intervention program is working from both the classroom teachers’ design of lessons and the students’ engagement with these activities—in different schools, with teachers drawn from different contexts, with varying teaching experience. It would be beneficial to capture changes in the classroom teachers’ discipline knowledge as they engage with the professional development (PD) aspects of the design and indeed as they implement the program. These data would allow for insights to be made about targeted PD—with those teachers with poor spatial reasoning skill afforded different levels of exposure to PD. The qualitative data provided promising insights into fidelity of the program; however, more systematic data should be collected on students’ reflections and engagement with the learning activities. Such qualitative depth will afford opportunities for researchers to monitor students’ sense making and skill development.

This investigation does not document learning transfer to other STEM fields, which should be examined in subsequent studies. The study was restricted to determining whether an integrated spatial thinking intervention program could improve student’s spatial thinking beyond a business-as-usual program that included spatial concepts embedded within the geometry and measurements mathematics program. Nevertheless, the study confirms that a classroom-based intervention program can improve student’s spatial reasoning, at scale. Large-scale, classroom-based, intervention programs can now be designed within attainable research budgets to determine transfer among STEM disciplines.

## Conclusions

This paper provides evidence for the effectiveness of a novel spatial reasoning intervention program, embedded within a pedagogical learning framework (ELPSA). There were statistically significant differences between the intervention and control groups, in favor of the intervention group. In addition, substantial gain score improvements were observed for all intervention students, irrespective of initial spatial reasoning performance. There was strong qualitative evidence that the classroom teachers introduced learning activities within the learning cycle and that students’ conceptual development followed this learning framework. Ultimately, this study shows promise for a spatial intervention that is effective in both enhancing spatial reasoning skills and inviting long-term engagement with the program.

## Additional files

Additional file 1: Spatial visualization lesson structured under the ELPSA framework: lines of symmetry lesson. (DOCX 673 kb)

Additional file 2: Data analysis using HLM. (DOCX 44 kb)

## Abbreviations

ACARA: Australian Curriculum, Assessment and Reporting Authority
ELPSA: Experience-Language-Pictorial-Symbolic-Application (learning framework)
SRI: Spatial Reasoning Instrument
STEM: Science, Technology, Engineering and Mathematics

## Footnotes

1 In Australia, the Index of Community Socio-Educational Advantage (ICSEA) is used to provide meaningful comparisons across schools. A score (mean = 1000, standard deviation = 100) is produced for each school, based on Australian Bureau of Statistics (ABS) data, school location, and the proportion of Indigenous students enrolled in the school. A value on the index corresponds to the average level of educational advantage of the school’s student population relative to those of other schools.

2 In Australia, the school year corresponds to the calendar year.
