# Supplementary material for: The impact of an intervention program on students’ spatial reasoning: student engagement through mathematics-enhanced learning activities
Source: Cogn Res Princ Implic. 2018 Dec 26;3:50. doi: 10.1186/s41235-018-0147-y (PMC6305681; doi:10.1186/s41235-018-0147-y)
Supplement: Supplementary file 1 — Spatial visualization lesson structured under the ELPSA framework: lines of symmetry lesson. (DOCX 673 kb) [file 41235_2018_147_MOESM1_ESM.docx]

**Additional file 1**

Spatial Visualization lesson structured under the ELPSA Framework – Lines of symmetry lesson

| ELPSA framework | Student Work samples | Student Voice |
| --- | --- | --- |
| **Experience:** What is symmetry?  Students begin with more familiar reflections of letters and symbols along the *y* and *x* axes. They are then asked to consider reflections of similar letters and symbols along the diagonal axis. | 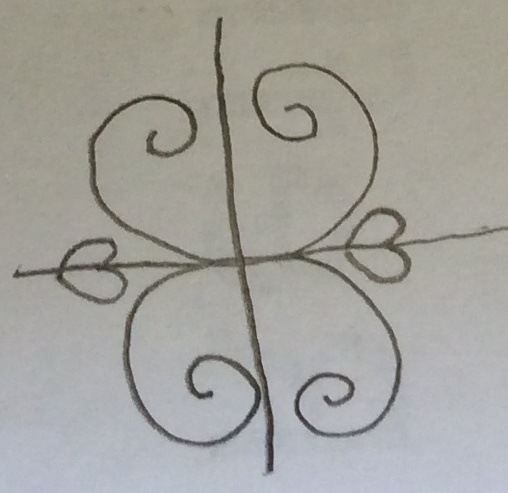 | “Symmetry is something like butterfly wings, they look the same on both sides and it might be a shape that is a mirror shape that is rotated” |
| **Language:** What are the language conventions associated with symmetry?  Line of symmetry; reflections; mirror image; visualize; fold; *y* and *x* axes; diagonal axis; vertical; horizontal;  imagine→predict→experiment→check; | 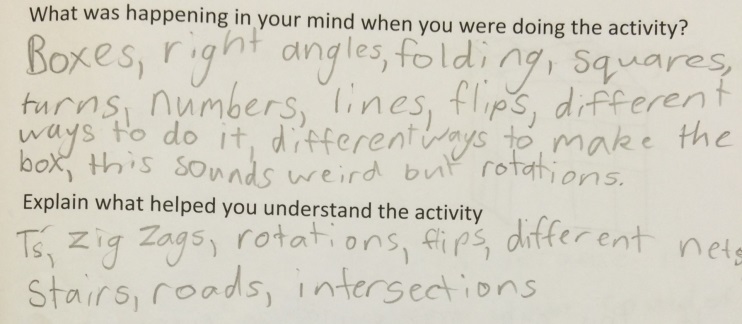 | “How to flip it on the y or x axis so I was trying to visualize the mirror”  “Today we revised on our spatial awareness. I understand now what the *y* and *x* axes are. The Y axis is when it goes these ways ↕ and the x axis when it goes these ways ↔” |
| **Pictorial:** Students represent their ideas about lines of symmetry in a drawing. They are required to draw a square with letters in the bottom left corner and a vertical (*y* axis) line of symmetry. Teacher example below. Move onto horizontal (*x* axis) line of symmetry and then diagonal line of symmetry.  **Pictorial (continued)**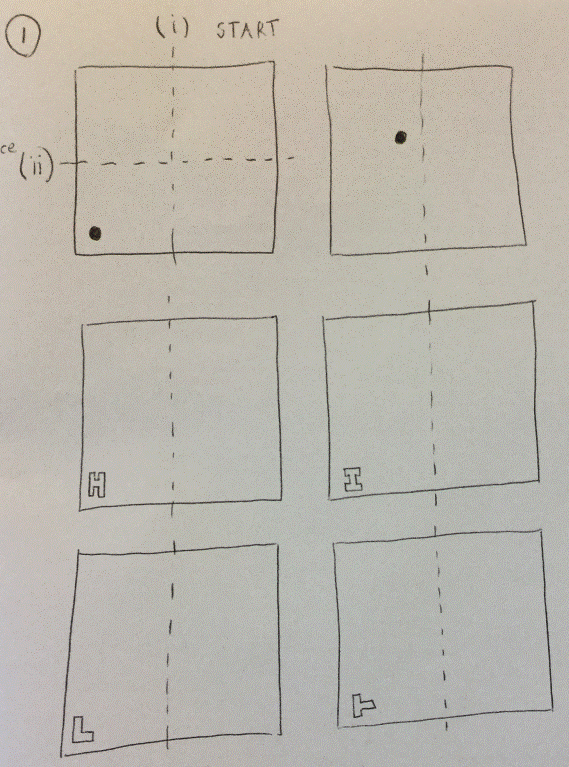 | 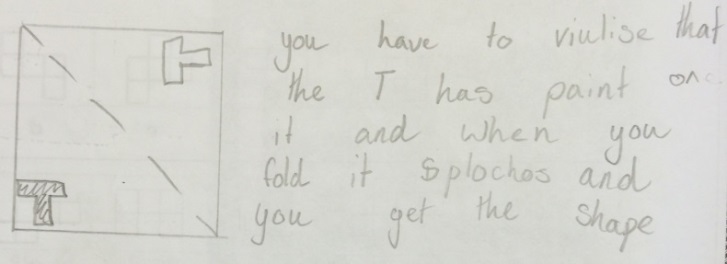  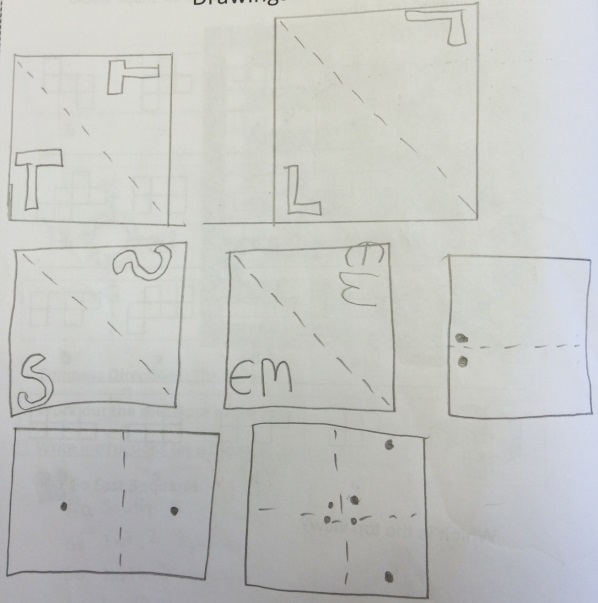 | “I was imagining a mirror reflecting the picture”  “I was imagining in my mind the paper folding and where it would print”  “I worked it out by picturing it in my mind and trying to think of the page was folded diagonally and thinking what the "T" was doing during that time”  “I was visualizing if I flipped it, what shape would it make and where would it be?”  “When I was doing this activity I was flipping the images in my mind to get the reflections correct. I was also imagining a mirror on the line so I could draw the reflections correctly.” |
| **Symbolic:** Symbolic stage requires analytic thinking. Here students need to recognize conventions associated with lines of reflection on vertical, horizontal and diagonal axes. Students begin to reason that for reflections on the *x* and *y* axes, horizontal stays horizontal and vertical stays vertical. However, with diagonal reflections, horizontal moves to vertical and vertical moves to horizontal. See images below for the concept of perpendicularity.  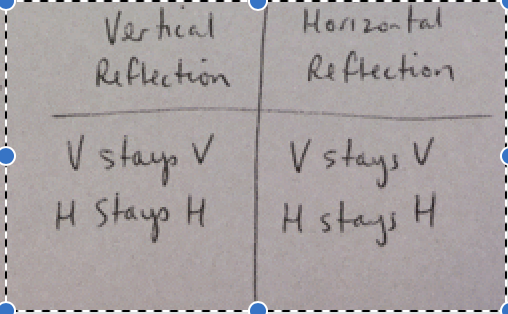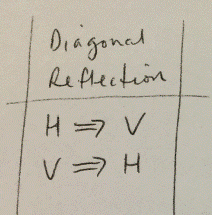 | 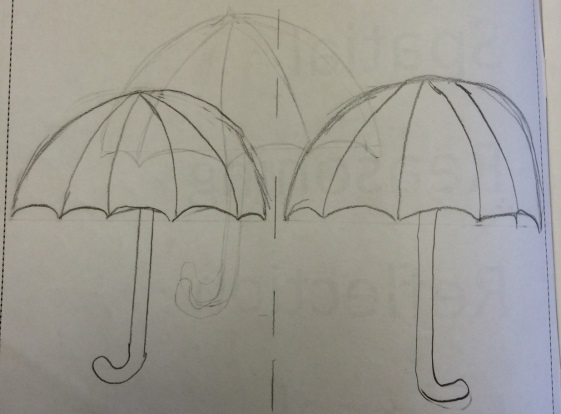 | “I did have to think a bit harder to remember that if the curve in the umbrella on the left is facing left, then the one on the right will be opposite.”  “I was imagining a mirror on the fold of the page. Using visual measurements to make it as accurate as possible”  “When I was doing the activity I remembered that we talked about the arrow and how it went from ↑ to ←.”  “I used my hand a first then I realised I could just reflect then rotate which was much easier.” (*diagonal reflection*) |
| **Application:** Application to other symmetry and problem solving tasks. | 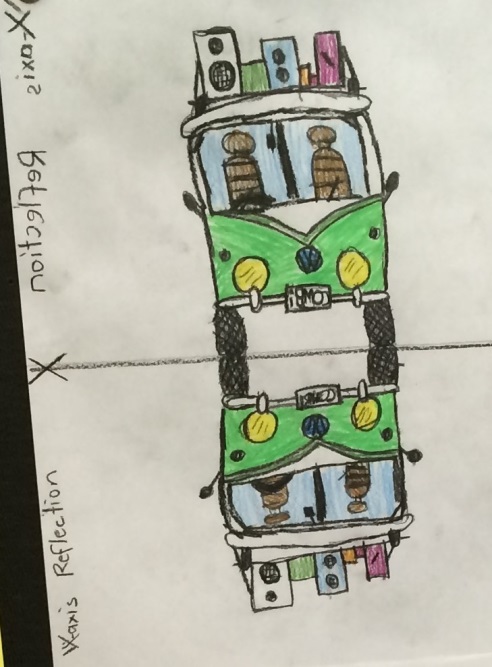 | This is a picture of a bus with a reflection (from water on the road) that considers both horizontal and vertical reflections. |
