# Supplementary material for: The impact of an intervention program on students’ spatial reasoning: student engagement through mathematics-enhanced learning activities
Source: Cogn Res Princ Implic. 2018 Dec 26;3:50. doi: 10.1186/s41235-018-0147-y (PMC6305681; doi:10.1186/s41235-018-0147-y)
Supplement: Supplementary file 2 — Data analysis using HLM. (DOCX 44 kb) [file 41235_2018_147_MOESM2_ESM.docx]

**Additional file 2**

**DATA ANALYSIS USING HLM**

- IMB SPSS Statistics (Version 24) was used to conduct the Hierarchical Linear Modelling (HLM).
- The analysis was conducted through the menu screen as follows: Analyze > Mixed Models > Linear.
- The variables in the analysis were as follows.
  - *Cohort:* (IV) [Treatment; Control]
  - *SRI Gain score* (DP) = SRI posttest – SRI pretest
- The analysis was undertaken with school class (class) nested. That is, subjects nested by class

*The following printout is produced.*

MIXED SRI_gain_score WITH cohort

/CRITERIA=CIN(95) MXITER(100) MXSTEP(10) SCORING(1) SINGULAR(0.000000000001) HCONVERGE(0,

ABSOLUTE) LCONVERGE(0, ABSOLUTE) PCONVERGE(0.000001, ABSOLUTE)

/FIXED=cohort | SSTYPE(3)

/METHOD=REML

/PRINT=DESCRIPTIVES SOLUTION

/RANDOM=INTERCEPT cohort | SUBJECT(class) COVTYPE(VC).

| **Descriptive Statistics** | | | | | |
| --- | --- | --- | --- | --- | --- |
| SRI_gain_score | | | | | |
| class | cohort | Count | Mean | Standard Deviation | Coefficient of Variation |
| 1 | Treatment | 27 | 4.74 | 3.612 | 76.2% |
| 2 | Treatment | 21 | 3.81 | 3.415 | 89.6% |
| 3 | Treatment | 34 | 4.24 | 3.172 | 74.9% |
| 4 | Treatment | 19 | 5.42 | 2.567 | 47.4% |
| 5 | Control | 24 | 1.04 | 2.881 | 276.6% |
| 6 | Control | 22 | 2.64 | 2.610 | 99.0% |
| 7 | Treatment | 32 | 3.22 | 3.230 | 100.4% |
| 8 | Control | 18 | 1.67 | 3.565 | 213.9% |
| 9 | Control | 20 | 2.55 | 4.097 | 160.7% |
| 10 | Control | 21 | 3.71 | 3.538 | 95.2% |
| 11 | Treatment | 21 | 3.14 | 3.151 | 100.3% |
| 12 | Treatment | 19 | 6.16 | 3.640 | 59.1% |
| 13 | Treatment | 20 | 3.80 | 2.895 | 76.2% |
| 14 | Control | 20 | 3.90 | 3.972 | 101.9% |
| 15 | Control | 19 | 1.37 | 3.166 | 231.4% |
| Total | Treatment | 193 | 4.23 | 3.317 | 78.4% |
|  | Control | 144 | 2.40 | 3.505 | 145.9% |
|  | Total | 337 | 3.45 | 3.512 | 101.8% |
|  | | | | | |

We analyzed group differences on pretests using a two-level HLM model (students within classrooms) with conditions dummy coded (1 = intervention and 0 = control). A two-level model was also used to analyze pretest-posttest gains; with condition groups similarly dummy coded to determine the direct effects of the interaction.

| **Model Dimension^a^** | | | | | |
| --- | --- | --- | --- | --- | --- |
|  | | Number of Levels | Covariance Structure | Number of Parameters | Subject Variables |
| Fixed Effects | Intercept | 1 |  | 1 |  |
|  | cohort | 1 |  | 1 |  |
| Random Effects | Intercept + cohort^b^ | 2 | Variance Components | 2 | class |
| Residual | |  |  | 1 |  |
| Total | | 4 |  | 5 |  |
| a. Dependent Variable: SRI_gain_score. | | | | | |
|  | | | | | |

| **Information Criteria^a^** | |
| --- | --- |
| -2 Restricted Log Likelihood | 1775.219 |
| Akaike's Information Criterion (AIC) | 1781.219 |
| Hurvich and Tsai's Criterion (AICC) | 1781.292 |
| Bozdogan's Criterion (CAIC) | 1795.662 |
| Schwarz's Bayesian Criterion (BIC) | 1792.662 |
| The information criteria are displayed in smaller-is-better form. | |
| a. Dependent Variable: SRI_gain_score. | |

Results from the hierarchical linear models for pretest-posttest gains revealed gain scores greater than 0 for each group across the two measures (see Table 3 for observed mean gains). The intercept slope was statistically significant *F*(1, 11) = 54.16, *p* < .001. On average, students in the intervention group gained 1.35 score points more than the control group on the SRI *t*(12) = 11.25, *p* < .001.

| **Type III Tests of Fixed Effects^a^** | | | | |
| --- | --- | --- | --- | --- |
| Source | Numerator df | Denominator df | F | Sig. |
| Intercept | 1 | 10.233 | 54.156 | .000 |
| cohort | 1 | 11.997 | 11.250 | .001 |
| a. Dependent Variable: SRI_gain_score. | | | | |

| **Estimates of Fixed Effects^a^** | | | | | | | |
| --- | --- | --- | --- | --- | --- | --- | --- |
| Parameter | Estimate | Std. Error | df | t | Sig. | 95% Confidence Interval | |
|  |  |  |  |  |  | Lower Bound | Upper Bound |
| Intercept | 6.147230 | .834956 | 10.233 | 7.362 | .000 | 4.292567 | 8.001893 |
| cohort | -1.868951 | .557214 | 11.997 | -3.354 | .001 | -3.083053 | -.654848 |
| a. Dependent Variable: SRI_gain_score. | | | | | | | |

| **Estimates of Covariance Parameters^a^** | | | |
| --- | --- | --- | --- |
| Parameter | | Estimate | Std. Error |
| Residual | | 11.009708 | .868316 |
| Intercept [subject = class] | Variance | .504330 | .804700 |
| cohort [subject = class] | Variance | .057311 | .308827 |
| a. Dependent Variable: SRI_gain_score. | | | |

Table 3

*Pretests, Posttests and Pretest-Posttest Gains for SRI by Condition*

|  | Intervention | | | | | | Control | | | | | |
| --- | --- | --- | --- | --- | --- | --- | --- | --- | --- | --- | --- | --- |
|  | Pretest | | Posttest | | Gain | | Pretest | | Posttest | | Gain | |
| Measure | *M* | *SD* | *M* | *SD* | *M* | *SD* | *M* | *SD* | *M* | *SD* | *M* | *SD* |
| SRI Perform | 15.24 | 4.75 | 19.48 | 5.51 | 4.42 | 4.95 | 15.09 | 4.77 | 18.76 | 5.41 | 3.67 | 4.95 |

| **Estimates of Covariance Parameters^a^** | | | | | | | |
| --- | --- | --- | --- | --- | --- | --- | --- |
| Parameter | | Estimate | Std. Error | Wald Z | Sig. | 95% Confidence Interval | |
|  |  |  |  |  |  | Lower Bound | Upper Bound |
| Residual | | 11.009025 | .868209 | 12.680 | .000 | 9.432357 | 12.849242 |
| cohort [subject = class] | Variance | .640210 | .456951 | 1.401 | .161 | .158043 | 2.593395 |
| a. Dependent Variable: SRI_gain_score. | | | | | | | |

MIXED SRI_gain_score BY cohort SRI_rank

/CRITERIA=CIN(95) MXITER(100) MXSTEP(10) SCORING(1) SINGULAR(0.000000000001) HCONVERGE(0,

ABSOLUTE) LCONVERGE(0, ABSOLUTE) PCONVERGE(0.000001, ABSOLUTE)

/FIXED=cohort SRI_rank cohort*SRI_rank | SSTYPE(3)

/METHOD=REML

/PRINT=DESCRIPTIVES

/RANDOM=SRI_rank cohort SRI_rank*cohort | SUBJECT(class) COVTYPE(VC).

| **Descriptive Statistics** | | | | |
| --- | --- | --- | --- | --- |
| Dependent Variable: SRI_gain_score | | | | |
| SRI_rank | cohort | Mean | Std. Deviation | N |
| 1 | Treatment | 4.52 | 3.164 | 60 |
|  | Control | 3.13 | 3.170 | 39 |
|  | Total | 3.97 | 3.167 | 99 |
| 2 | Treatment | 4.27 | 3.472 | 80 |
|  | Control | 2.42 | 3.112 | 62 |
|  | Total | 3.21 | 3.264 | 142 |
| 3 | Treatment | 4.23 | 3.311 | 53 |
|  | Control | 2.90 | 3.418 | 43 |
|  | Total | 3.36 | 3.327 | 86 |
| Total | Treatment | 4.34 | 3.317 | 193 |
|  | Control | 2.81 | 3.305 | 144 |
|  | Total | 3.64 | 3.212 | 337 |

| **Model Dimension^a^** | | | | | |
| --- | --- | --- | --- | --- | --- |
|  | | Number of Levels | Covariance Structure | Number of Parameters | Subject Variables |
| Fixed Effects | Intercept | 1 |  | 1 |  |
|  | cohort | 2 |  | 1 |  |
|  | SRI_rank | 3 |  | 2 |  |
|  | cohort * SRI_rank | 6 |  | 2 |  |
| Random Effects | SRI_rank + cohort + cohort * SRI_rank^b^ | 11 | Variance Components | 3 | class |
| Residual | |  |  | 1 |  |
| Total | | 23 |  | 10 |  |
| a. Dependent Variable: SRI_gain_score. | | | | | |
|  | | | | | |

| **Between-Subjects Factors** | | | |
| --- | --- | --- | --- |
|  | | Value Label | N |
| SRI_rank | 1 |  | 99 |
|  | 2 |  | 142 |
|  | 3 |  | 86 |
| cohort | 1.00 | Treatment | 193 |
|  | 2.00 | Control | 144 |

| **Type III Tests of Fixed Effects^a^** | | | | |
| --- | --- | --- | --- | --- |
| Source | Numerator df | Denominator df | F | Sig. |
| Intercept | 1 | 13.901 | 144.404 | .000 |
| cohort | 1 | 13.901 | 7.851 | .014 |
| SRI_rank | 2 | 327.305 | 7.688 | .001 |
| cohort * SRI_rank | 2 | 327.305 | 1.378 | .253 |
| a. Dependent Variable: SRI_gain_score. | | | | |

To determine the extent to which the intervention program advantaged students of varying levels of initial spatial reasoning performance, a subsequent analysis was undertaken to determine whether there was an interaction effect between student’s initial spatial reasoning skill and their post SRI scores. Students’ initial SRI scores were coded as low, medium or high where low equalled the lowest quartile, high equalled the highest quartile, and medium the remaining fifty per cent. Gain scores were generated for the respective spatial ranks. Results from the hierarchical linear model gain scores greater than 0 for each group across the two measures (see Table 4 for observed mean gains). The 2 (control v intervention) x 3 (low, medium, high) ANOVA revealed no statistical significant interaction between cohort and initial rank on the SRI *F*(2, 14) = 1.38, *p* >.05.

Effect sizes (Cohen’s *d*) between intervention and control group SRI gain scores by spatial rank are presented in Table 4. We can conclude that the spatial reasoning program was beneficial for the intervention group irrespective of their initial spatial reasoning skill.

Table 4 *SRI Gain Scores (Mean (S.D.)) by Spatial Rank for Each Cohort*

| Spatial Rank | Intervention | Control | Effect size  (*d*) |
| --- | --- | --- | --- |
| Low | 4.52 (3.16) | 3.13 (3.22) | .44 |
| Medium | 4.27 (3.47) | 2.42 (3.11) | .56 |
| High | 4.23 (3.31) | 2.90 (3.41) | .43 |

Note: Intervention Low N = 60, Medium N = 80, High N = 53. Control Low N = 39, Medium N = 62, High N = 43.
